# A Modified Bayesian Framework for Multi-Sensor Target Tracking with Out-of-Sequence-Measurements

**DOI:** 10.3390/s20143821

**Published:** 2020-07-09

**Authors:** Yifang Shi, Sundas Qayyum, Sufyan Ali Memon, Uzair Khan, Junaid Imtiaz, Ihsan Ullah, Darren Dancey, Raheel Nawaz

**Affiliations:** 1 School of Automation, Hangzhou Dianzi University, Hangzhou 310018, China; syf2008@hdu.edu.cn; 2 Department of Electrical and Computer Engineering, CUI, Abbottabad Campus, Abbottabad 22060, Pakistan; sundasqayyum@uiatd.edu.pk (S.Q.); uzairkhan@cuiatd.edu.pk (U.K.); 3 Department of Mechatronics Engineering, Mehran University, Jamshoro 76090, Pakistan; sufyan.memon@faculty.muet.edu.pk; 4 Department of Electrical Engineering, Bahria University Islamabad Campus, Islamabad 44230, Pakistan; junaidimtiaz.buic@bahria.edu.pk; 5 School of Computer Science, Manchester Metropolitan University, Manchester M15 6BH, UK; d.dancey@mmu.ac.uk (D.D.); r.nawaz@mmu.ac.uk (R.N.)

**Keywords:** tracking, estimation, OOSM, false track discrimination, sensor fusion

## Abstract

Target detection and tracking is important in military as well as in civilian applications. In order to detect and track high-speed incoming threats, modern surveillance systems are equipped with multiple sensors to overcome the limitations of single-sensor based tracking systems. This research proposes the use of information from RADAR and Infrared sensors (IR) for tracking and estimating target state dynamics. A new technique is developed for information fusion of the two sensors in a way that enhances performance of the data association algorithm. The measurement acquisition and processing time of these sensors is not the same; consequently the fusion center measurements arrive out of sequence. To ensure the practicality of system, proposed algorithm compensates the Out of Sequence Measurements (OOSMs) in cluttered environment. This is achieved by a novel algorithm which incorporates a retrodiction based approach to compensate the effects of OOSMs in a modified Bayesian technique. The proposed modification includes a new gating strategy to fuse and select measurements from two sensors which originate from the same target. The state estimation performance is evaluated in terms of Root Mean Squared Error (RMSE) for both position and velocity, whereas, track retention statistics are evaluated to gauge the performance of the proposed tracking algorithm. The results clearly show that the proposed technique improves track retention and and false track discrimination (FTD).

## 1. Introduction

Detection and tracking of high-speed targets for interception or early warning is a challenging problem [1]. Several types of sensors like RADAR, IR, LADAR are used to detect or engage any threats. Some tracking techniques employ a single sensor, while, others may use a combination of two or more sensors (depending on the application) that may or may not be similar. In order to obtain information about targets dynamics most single sensor-based tracking systems rely on a RADAR. A RADAR typically has high precision range measurement and narrow beam width. Alongside the range, an active RADAR also measures the azimuth and elevation angles of the target. Due to the active nature of a RADAR, it can easily be jammed or face performance degradation with Electronic Counter Measures (ECM) deployed by an adversary. The weakness of a RADAR based system against ECM and the possibility of being detected due to its active nature, limit its use in many defense applications [2,3]. Moreover, another drawback of radar based tracking systems is the course angular (azimuth and elevation) measurements as compared to certain types of passive sensors.

An IR sensor is passive in nature, hence it is immune to electromagnetic interference [2,3,4]. However, it is quite sensitive to atmospheric conditions, which, limits its range but the angular measurements are more precise as compared to RADAR. Owing to this passive nature the range information (of target) is not provided by single IR sensor. Tracking accuracy of a system can be improved by using multiple sensors, along with providing redundancy to the tracking system [5]. Due to the complementary characteristics of RADAR /IR, considerable research is done on their fusion. In References [6,7], RADAR /IR fusion algorithms are discussed, fusion of these two sensors improves the estimation accuracy. In Reference [6], authors compare two measurement fusion techniques (RADAR/IR) for 3D target tracking but this study is limited to a non-cluttered environment. In Reference [7], several multi sensor data fusion algorithms are discussed which are based on KF based techniques in a non-cluttered environment. Some fusion algorithms with application to the multiple passive sensor scenario with same characteristics are discussed in References [8,9,10,11,12]; whereas complementary characteristics of LADAR/IR fusion is discussed in References [13,14]. Due to passive sensing, multiple sensors are necessary to track a target in 3D, these sensors should be separated by a certain baseline distance. The greater the baseline distance the greater will be the detection range. The baseline distance makes this approach only suitable for close range targets, generally used in civilian applications such as autonomous vehicle navigation or close-in weapon systems [15,16].

**Figure 1:**
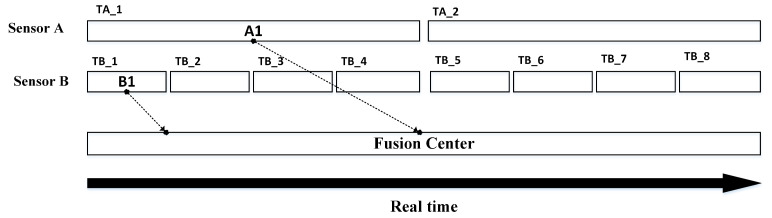
Out-of-sequence measurements (OOSMs) Scenario with different measurement arrival time.

While dealing with the problem of multiple sensor fusion with complementary characteristics, each sensor has different processing time as well as measurement acquisition rate, thus the measurements may arrive at processing unit out of sequence [17], as depicted in Figure 1. In a realistic tracking scenario, multiple measurements may be received due to cluttered environment. The clutter measurements originate random sources other than target generated detections [18]. The issue related to clutter can be resolved by a data association algorithm such as Integrated Probabilistic Data Association (IPDA) [19]. It is implemented recursively and as a track quality measure, the Probability of Track Existence (PTE) is estimated which makes it superior to other probabilistic data association algorithms [20]. A feature known as False Track Discrimination (FTD) is used in IPDA for confirmation and termination of tracks [21].

Various techniques have been used in literature to solve the OOSM problem. One approach [22] is to reprocess the OOSM measurements, where the target state estimate, sensor measurements and associated error covariances are stored until the delayed measurement is detected. This approach requires significant computer resources for data storage and filtering of measurements, but its estimation accuracy is high, as for orderly measurements Kalman Filter (KF) is used. In another approach [23], a buffer (of size greater than the maximum expected delay) is used to store the incoming measurements. These time-ordered measurements are extracted from the buffer upon arrival of an OOSM for filtering process. This approach requires significant memory, and storage management also needs to be considered; however, re-filtering is not required, since all information is filtered once the delayed measurement has arrived. This approach is unsuitable for real-time applications because filtering process lags behind the current time. Discarding of time delayed data is another approach, when the central processor receives a delayed measurement it is simply discarded. It is the simplest approach and requires the least amount of hardware resources, however, the estimation accuracy is compromised, especially when the quantity of these delayed measurements is significant.

In most of the cases, OOSM estimation techniques use a retrodiction based approach, that is, the current state is predicted backwards to the originating time of the measurement which is now out of sequence. Such an approach is proposed in Reference [24], which compresses the information of the updates into a single one by updating the estimate between $t_{K-d}$ to $t_{k}$, where, $t_{k}$ represents the current time and $t_{k-d}$ denotes the OOSM arrival time. The proposed approach is an optimal one, but it works when the in-sequence measurements arriving prior to the OOSM, are both in sequence and consecutive. Reference [24] also provides a suboptimal solution for this particular problem and is referred to as the B1 algorithm. A new algorithm based on the framework of B1 algorithm is proposed in Reference [25]. The proposed algorithm provides a solution for the l-lag OOSM problem, where the associated covariances are stored for filter gain computation from the past sampling intervals. A one step solution to multiple lag OOSM problem is proposed in Reference [26], the authors proposed two algorithms with one being optimal and the other one suboptimal, known as Al1 and Bl1 algorithms, respectively. The aforementioned OOSM filtering algorithms can incorporate single as well multiple-lag OOSMs with a considerable reduction in estimation error and roughly are based on the framework of A1 and B1 algorithms. The Bl1 algorithm was incorporated in the proposed research because of its ability to solve multiple lag OOSMs in one step.

There are other techniques discussed in References [25,26,27] which require more memory and computational resources, making them impractical for real time applications, another issue with these techniques is that they are only applicable in non-cluttered environment. The result section shows a comparison of the proposed algorithm with algorithm presented in Reference [3], which uses the NN technique to resolve the clutter problem but considers measurement origination from target only for the supplementary sensor. Several algorithms for OOSM update are presented in References [28,29,30], in which pseudo measurements, Kalman filtering, optimal and sub-optimal approaches are discussed, however, these approaches solve the problem of OOSMs in non-cluttered environment.

Data association algorithms are used in cluttered environment, where measurement not only originate from the target but also from other sources (thermal noise, obstacles, clouds, terrain) [11,31]. A Bayesian data association technique, Probabilistic data association (PDA), uses all the latest validated measurements with different weights for associating any validated measurements with the track [32]. It is a proven single target tracking algorithm in cluttered environment [33], however, at each scan it assumes track existence with a probability of one, that is, target existence information is not considered, in reality target may or may not exist. A novel algorithm, built around the PDA filter framework was proposed in References [34,35] known as the IPDA. It re-derives the PDA algorithm without any initial assumption of track existence and provides expressions for both PTE and data association probability, simultaneously and recursively. These techniques have been developed in the literature, with some of them specifically designed for a non-cluttered environemnt whereas others for cluttered environment.

The authors were not able to find any algorithm which solved the OOSM fusion problem for multiple sensors with complimentary characteristics in cluttered environment using a Bayesian framework with FTD. So, there exists a gap in the literature which is of significant value and has a broad range of applications. This research tries to narrow down this gap by proposing a novel algorithm for multiple sensor data fusion in cluttered environment. The algorithm is further modified to incorporate the OOSM scenarios. The resulting algorithm is built around the IPDA filter framework and is capable of tracking single target for OOSM case in cluttered environment. A novel angle on gating strategy is used for the OOSMs from the IR sensor by using estimates from the existing tracks only. The pseudo measurements are generated and are followed by a NN method of measurement selection for further processing. This is fed to the track maintenance algorithm thus refining the overall target trajectory performance. The performance analysis proves that the proposed algorithm is superior in terms of RMSE of the estimated target dynamics and FTD, when, compared to the single sensor case as well as multiple sensor case with OOSM [3].

Section 2 provides the details about the proposed framework and mathematical modeling. Detailed simulations were carried out for performance evaluation of the proposed algorithm with and without OOSMs and are discussed in Section 3 along with the comparison of proposed techniques with single sensor approach. Section 4 provides results and discussion which is followed by the conclusion in Section 5.

## 2. Modified Bayesian Algorithm for OOSM Incorporation

**Figure 2:**
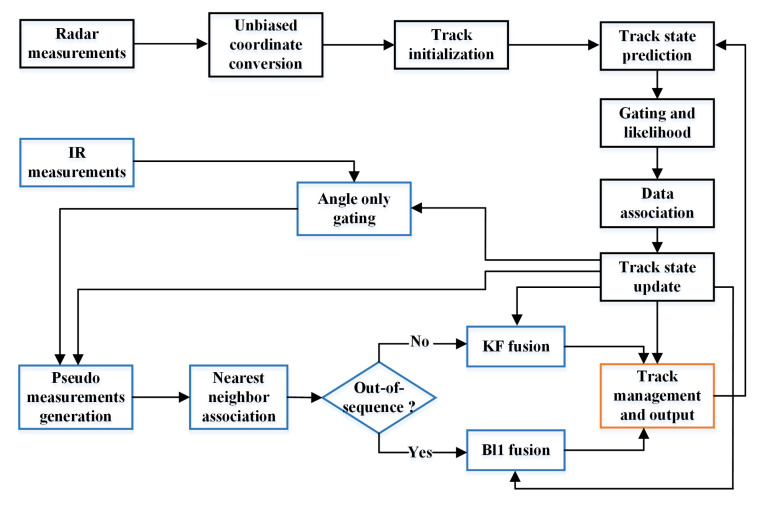
Block diagram of the Proposed algorithm.

Figure 2 shows block diagram of the proposed algorithm, as can be seen there, the proposed algorithm consists of three parts: firstly, employ the integrated probabilistic data association (IPDA) approach for radar tracking in the presence of target mis-detection and clutter disturbance, as a result, a set of radar-updated tracks are obtained;then, an angle only gating technique is carried out to select a subset of IR measurements based on the radar-updated track estimates, which are used to generate a set of pseudo measurement aimed at eliminating bias, then the nearest neighbor technique is deployed to associate a feasible pseudo measurement to be fused with the radar-updated track state; at last, the track management procedure is implemented by using the fused PTE as a track quality measure and output the target kinematic state estimates.

### 2.1. System Models

This paper considers the incoming target tracking in the environment of target mis-detection and clutter disturbance using multiple heterogeneous sensor information. The multi-sensor information concerns here are mainly referred to the position measurements from radar and angle measurements from IR sensor, with the IR sensor data potentially arriving at the fusion center with out of temporal sequence. In order to focus on the main tracking challenges, the targets considered here are assumed to be point target, and both the radar and IR sensor are assumed to be with infinite sensor resolution. The necessary system models are mathematically formulated in this section.

#### 2.1.1. Target Model

The target randomly appears and disappears in the surveillance space, consequently, its existence is a random event and modeled by a binary random variable. Denoting the target existences at time $t_{k}$ by $\chi_{k}$, which evolves as a first order Markov Chain in the time domain, and the probability that the target exists at time $t_{k}$ conditioned on it did exist at time $t_{k-1}$ is mathematically described by

(1) $$p_{11}=P\left(\chi_{k}|\chi_{k-1}\right)\approx 1-\frac{\Delta T_{k,k-1}}{T_{ave}},$$

where $\Delta T_{k,k-1}$ is the time interval of two consecutive scans, $T_{ave}$ denotes the average target existence duration and usually $T_{ave}>>T_{k,k-1}$. In this paper, that the possibility of target birth has been treated by the random track initialization procedure, the probability that target exists at time $t_{k}$ given that it did not exist at time $t_{k-1}$ is assumed to be zero, that is,

(2) $$p_{12}=P\left(\chi_{k}|{\bar{\chi }}_{k-1}\right)=0.$$

Once the target exists in the surveillance area, its kinematic state needs to be estimated. For the sake of simplicity and clarity, the dynamic model of the target of interest is assumed to be linear and described by

(3) $$x_{k}=F_{k,k-1}x_{k-1}+w_{k},$$

where the target kinematic state consists of the 3D position and velocity, that is, $x_{k}={\left[x_{k}\;y_{k}\;z_{k}\;{\dot{x}}_{k}\;{\dot{y}}_{k}\;{\dot{z}}_{k}\right]}^{T}$, and $w_{k}$ is the process noise, which is modeled by the additive white Gaussian noise, with zero mean and covariance $Q_{k,k-1}$,

(4) $$Q_{k,k-1}=q\left[\begin{array}{cc} \frac{T_{k,k-1}^{3}}{3} & \frac{T_{k,k-1}^{2}}{2}\\ \frac{T_{k,k-1}^{2}}{2} & T_{k,k-1} \end{array}\right]⊗I_{3},$$

where *q* denotes the power spectral density, ⊗ is the Kronecker product, $I_{3}$ is the 3D identity matrix. $F_{k,k-1}$ denotes the dynamic state transition matrix from time $t_{k-1}$ to $t_{k}$, and given as

(5) $$F_{k,k-1}=\left[\begin{array}{cc} 1 & \Delta T_{k,k-1}\\ 0 & 1 \end{array}\right]⊗I_{3}.$$

The target state $\left(\chi_{k},x_{k}\right)$ modeled above is able to fully describe the statistics of the target behavior, wherein, the probability of target existence $\chi_{k}$ is used as an efficient track quality measure for track management, the kinematic state $x_{k}$ is only defined conditioning on the target existence $\chi_{k}$.

#### 2.1.2. Sensors Model

At each time, both the radar and IR sensor receive a set of origin-unknown measurements, with their cardinality randomly varying. Let $Z_{k}^{s}$ and $Z^{k,s}$ denote the set of measurements collected by sensor *s* at time $t_{k}$, up to and including time $t_{k}$, respectively, with s=r denoting radar returned measurements, and s=IR referring to IR returned measurements. Denoting the *i*th measurement of $Z_{k}^{s}$ by $Z_{k,i}^{s}$. Due to imperfect detection, either radar or IR returns the target measurement with a detection probability $P_{D}$. At time $t_{k}$, the radar measures the target range $r_{k}$, azimuth $\eta_{k}^{r}$ and elevation $\varepsilon_{k}^{r}$ in the cylindrical coordinate, which is a nonlinear function of the kinematic states of both the target and radar,

(6) $$\begin{array}{c} z_{k}^{r}={\left[r_{k}\;\;\;\;\eta_{k}^{r}\;\;\;\;\varepsilon_{k}^{r}\right]}^{T}=h^{r}\left(x_{k},p_{k}^{r}\right)+v_{k}^{r}=\left[\begin{array}{c} \sqrt{{\left(x_{k}-x_{k}^{r}\right)}^{2}+{\left(y_{k}-y_{k}^{r}\right)}^{2}+{\left(z_{k}-z_{k}^{r}\right)}^{2}}\\ \tan^{-1}\frac{y_{k}-y_{k}^{r}}{x_{k}-x_{k}^{r}}\\ \tan^{-1}\frac{z_{k}-z_{k}^{r}}{\sqrt{{\left(x_{k}-x_{k}^{r}\right)}^{2}+{\left(y_{k}-y_{k}^{r}\right)}^{2}}} \end{array}\right]+\left[\begin{array}{c} v_{k}^{r}\\ v_{k}^{\eta_{r}}\\ v_{k}^{\varepsilon_{r}} \end{array}\right], \end{array}$$

where $p_{k}^{r}$ is the radar position vector at time $t_{k}$, $v_{k}^{r}$ denotes the radar measurement noise, which is described as an additive white Gaussian, with zero mean and known covariance $R_{k}^{r}$. At time $t_{k}$, the IR senor can only measure the angle information of target, that is, azimuth $\eta_{k}^{IR}$ and elevation $\varepsilon_{k}^{IR}$, which is also a nonlinear function of the kinematic states of both the target and radar,

(7) $$\begin{array}{c} z_{k}^{IR}={\left[\eta_{k}^{IR}\;\;\;\;\varepsilon_{k}^{IR}\right]}^{T}=h^{IR}\left(x_{k},p_{k}^{IR}\right)+v_{k}^{IR}=\left[\begin{array}{c} \tan^{-1}\frac{y_{k}-y_{k}^{IR}}{x_{k}-x_{k}^{IR}}\\ \tan^{-1}\frac{z_{k}-z_{k}^{IR}}{\sqrt{{\left(x_{k}-x_{k}^{IR}\right)}^{2}+{\left(y_{k}-y_{k}^{IR}\right)}^{2}}} \end{array}\right]+\left[\begin{array}{c} v_{k}^{\eta_{IR}}\\ v_{k}^{\varepsilon_{IR}} \end{array}\right], \end{array}$$

where $p_{k}^{IR}$ denotes the IR sensor position vector at time $t_{k}$, $v_{k}^{IR}$ is the IR measurement noise, which is described as an additive white Gaussian, with zero mean and known covariance $R_{k}^{IR}$. Apart from target measurement, both the radar and IR sensor also return a set of clutter measurements originated from either unwanted targets or thermal noise. The number of clutter measurements at each time $t_{k}$ is random and follows a Poisson distribution, the intensity of each clutter measurement $Z_{k,i}^{s}$ in the surveillance is termed as clutter measurement density and denoted by $\rho (Z_{k,i}^{s})$, which is usually assumed to be known or estimated.

The main concern of this paper is to fuse multiple source information to achieve an improved tracking performance, that is, to obtain the posterior estimates of the target state $p(\chi_{k},x_{k}|Z^{k,r},Z^{l,IR})$ based on measurements collecting from radar and IR at time $t_{k}$, in which the IR measurements $Z^{l,IR}$ may arrive at the fusion center with out of temporal sequence ( $t_{l}<t_{k}$).

### 2.2. Radar Tracking Using the Ipda Algorithm

Since radar is able to measure the complement position measurement (i.e., range, azimuth and elevation) of targets of interest, the integrated probabilistic data association (IPDA) is utilized here for radar tracking to estimate the target state in the presence of target mis-detection and clutter disturbance. In order to reduce the estimation bias, the nonlinear radar measurements in cylindrical coordinate are converted to the Cartesian coordinate. Based on the unbiased conversion method proposed in Reference [36], the radar cylindrical measurement $z_{k}^{r}={\left[r_{k}\;\eta_{k}^{r}\;\varepsilon_{k}^{r}\right]}^{T}$ is converted to the position measurement in the 3D Cartesian coordinate, that is, $z_{k}^{r}={\left[x_{k}^{r}\;y_{k}^{r}\;z_{k}^{r}\right]}^{T}$, with its corresponded measurement noise $R_{k}^{r,p}$, the detailed calculation to obtain them, please refer to Reference [36].

The IPDA algorithm recursively updates the target state estimate based on system models defined in last subsection and radar measurements received at time $t_{k}$. The target state $\left(\chi_{k-1},x_{k-1}\right)$ at time $t_{k-1}$ is mathematically described by a posterior probability density function (pdf) $p(\chi_{k-1},x_{k-1}|Z^{k-1,r})$, consisting of the probability of target existence $p(\chi_{k-1}|Z^{k-1,r})$ and the posterior pdf of the target kinematic state $p(x_{k-1}|\chi_{k-1},Z^{k-1,r})$ at time $t_{k-1}$. For simplicity, in the rest of this paper, the pdf of target kinematic state is implicitly conditioned on the target existence, that is, $p\left(x_{k-1}|\chi_{k-1},Z^{k-1}\right)\equiv p\left(x_{k-1}|Z^{k-1,r}\right)$.

(8) $$p\left(\chi_{k-1},x_{k-1}|Z^{k-1,r}\right)=p\left(x_{k-1}|Z^{k-1,r}\right)P\left(\chi_{k-1}|Z^{k-1,r}\right),$$

with the posterior pdf of kinematic state at time $t_{k-1}$ approximated by a Gaussian,

(9) $$p\left(x_{k-1}|Z^{k-1,r}\right)\approx N\left(x_{k-1};{\hat{x}}_{k-1|k-1},P_{k-1|k-1}\right).$$

In order to start the tracking recursion, the two-pointing differencing is implemented for initialize tentative tracks at every two scan using effective radar measurements that satisfy the maximum target moving velocity constraint. The probability of target existence of tentative track is initialized by given a small positive value, that is, $P\left(\chi_{0}|Z^{0,r}\right)=\psi_{0}$, and its initialized kinematic state is assumed to be a Gaussian, that is, $p\left(x_{0}|Z^{0,r}\right)\approx N\left(x_{0};{\hat{x}}_{0|0},P_{0|0}\right)$, with its mean and covariance calculated by

(10) $${\hat{x}}_{0|0}={\left[\begin{array}{c} \;\;\;\;\;\;\;\;\;\;\;\;z_{k}^{r}\;\;\;\;\\ \frac{z_{k}^{r}-z_{k-1}^{r}}{\Delta T_{k,k-1}} \end{array}\right]}^{T},$$

(11) $$P_{0|0}=\left[\begin{array}{c} \;\;\;\;\;R_{k}^{r,p}\;\;\;\;\;\;\;\;\;\;\;\;\;\;\;\;\;\;\;\;\;\;\;\;\frac{R_{k}^{r,p}}{\Delta T_{k,k-1}}\\ \frac{R_{k}^{r,p}}{\Delta T_{k,k-1}}\;\;\;\;\;\;\;\;\;\;\;\;\frac{2R_{k}^{r,p}}{{\left(\Delta T_{k,k-1}\right)}^{2}} \end{array}\right].$$

One IPDA tracking recursion usually consists of track state prediction, gating and likelihood, data association, track state update, each of them is introduced in detail at the rest of this subsection.

#### 2.2.1. Track State Prediction

The predicted track state at time $t_{k}$ consists of two parts

(12) $$p\left(\chi_{k},x_{k}|Z^{k-1,r}\right)=p\left(x_{k}|Z^{k-1,r}\right)P\left(\chi_{k}|Z^{k-1,r}\right),$$

where the predicted probability of target existence is obtained by

(13) $$P\left(\chi_{k}|Z^{k-1,r}\right)=p_{11}P\left(\chi_{k-1}|Z^{k-1,r}\right),$$

and the predicted target kinematic state pdf is described by a Gaussian, that is, $p\left(x_{k}|Z^{k-1}\right)=N\left(x_{k};{\hat{x}}_{k|k-1},P_{k|k-1}\right)$, with its mean and corresponded error covariance calculated by

(14) $$\left[{\hat{x}}_{k|k-1},\;\;\;\;\;\;P_{k|k-1}\right]\;=\;KF_{P}\left({\hat{x}}_{k-1|k-1},\;\;P_{k-1|k-1},F_{k,k-1},\;\;\;\;Q_{k,k-1}\right),$$

where $KF_{P}$ denotes the standard prediction process of Kalman filter.

#### 2.2.2. Gating and Likelihood

In order to release computation and storage burden, an ellipsoid gating technique is utilized to select a subset of feasible radar measurements for track update, that is,

(15) $${\left(z_{k,i}^{r}-h\left({\hat{x}}_{k|k-1},p_{k}^{r}\right)\right)}^{T}{\left(S_{k}\right)}^{-1}\left(z_{k,i}^{r}-h\left({\hat{x}}_{k|k-1},p_{k}^{r}\right)\right)\leq g,$$

with the innovation covariance equals

(16) $$S_{k}=H_{k}P_{k|k-1}H_{k}^{T}+R_{k}^{r,p},$$

where $H_{k}$ denotes the measurement Jacobian matrix evaluated at the ${\hat{x}}_{k|k-1}$, *g* is a gating threshold predefined based on the probability that measurements will lie inside the ellipsoid validation gate. Consequently, a subset of radar measurements $Z_{k}^{r}$ at time $t_{k}$ is selected and denoted by $z_{k}^{r}$, with its cardinality $m_{k}$. The likelihood of *i*th measurement of $z_{k}^{r}$ is thus calculated by

(17) $$p_{k,i}=p\left(z_{k,i}|Z^{k-1,r}\right)=\frac{N\left(z_{k,i};h^{r}\left({\hat{x}}_{k|k-1},p_{k}^{r}\right),S_{k}\right)}{P_{G}},$$

where $P_{G}$ is the gating probability that measurements will lie inside the validation gate.

#### 2.2.3. Data Association

Origins of the set of validated measurements $z_{k}^{r}$ is unknown, either from targets of interest or from clutter. Therefore, one needs to enumerate and evaluate all possibilities that validated measurements originate from targets. Let $\theta_{k,i},i\geq 0$ denote a measurement-to-target association event at time $t_{k}$, with i=0 denoting none of $z_{k}^{r}$ originates from targets, i>0 denoting the *i*th measurement of $z_{k}^{r}$ is the detection of target at time $t_{k}$. Denoting the posterior probability of association event $\theta_{k,i}$ conditioned on the target existence at time $t_{k}$ by $\beta_{k,i}$, which is calculated by [19],

(18) $$\beta_{k,i}\equiv P\left(\theta_{k,i}|\chi_{k},Z^{k,r}\right)=\frac{1}{\delta_{k}}\left\{\begin{array}{c} \frac{P_{D}P_{G}p_{k,i}}{\rho_{k,i}},\;\;\;\;\;\;\;\;\;\;\;\;\;\;\;\;\;\;i>0\\ 1-P_{D}P_{G}\;\;\;\;\;\;\;\;\;\;\;\;\;\;\;\;i=0, \end{array}\right.$$

with the likelihood ratio calculated by

(19) $$\delta_{k}=1-P_{D}P_{G}+P_{D}P_{G}\sum_{i=1}^{m_{k}}\frac{p_{k,i}}{\rho_{k,i}}.$$

#### 2.2.4. Track State Update

The updated track state estimate can be decomposed into two parts,

(20) $$p\left(\chi_{k},x_{k}|Z^{k,r}\right)=p\left(x_{k}|Z^{k,r}\right)P\left(\chi_{k}|Z^{k,r}\right),$$

where the updated probability of target existence is calculated by

(21) $$P\left(\chi_{k}|Z^{k,r}\right)=\frac{\delta_{k}P\left(\chi_{k}|Z^{k-1,r}\right)}{1-\left(1-\delta_{k}\right)P\left(\chi_{k}|Z^{k-1,r}\right)},$$

and the updated kinematic state pdf is represented by a single Gaussian, that is, $p\left(x_{k}|Z^{k,r}\right)\approx N\left(x_{k};{\hat{x}}_{k|k},P_{k|k}\right)$, which is a Gaussian mixture of kinematic state estimates updated using the validated measurements $z_{k}^{r}$,

(22) $$\left[{\hat{x}}_{k|k},P_{k|k}\right]=\mathrm{Gmix}{\left({\hat{x}}_{k|k,i},P_{k|k,i},\beta_{k,i}\right)}_{i=0}^{m_{k}},$$

where Gmix denotes the standard Gaussian mixture operation. The mean and corresponding error covariance of the kinematic state estimate updated using $z_{k,i}^{r}$ is obtained by

(23) $$\left[{\hat{x}}_{k|k,i},P_{k|k,i}\right]=KF_{U}\left({\hat{x}}_{k|k-1},P_{k|k-1},z_{k,i}^{r},R_{k,i}^{P},H_{k}\right),$$

where $KF_{U}$ is the standard update process of Kalman filter. As a consequence, the output of radar tracking at time $t_{k}$ is a set of updated track estimates, with each track state estimate including updated PTE and the posterior pdf of kinematic state represented by a Gaussian, that is, $\left\{P\left(\chi_{k}|Z^{k,r}\right),\;\;\left({\hat{x}}_{k|k},P_{k|k}\right)\right\}$.

### 2.3. Track Fusion with IR Information

The track estimates already updated using radar measurements $Z^{k,r}$ at time $t_{k}$ is then fused with the IR information $Z^{l,IR}$ received in the fusion center, with time stamp $t_{l}\leq t_{k}$, aimed at further improving the tracking results. When $t_{l}=t_{k}$, it means the IR information arrives at the fusion center with in sequence temporal order and becomes an in-sequence measurements (ISMs) fusion problem, while, when $t_{l}<t_{k}$, the IR information arrives at fusion center with out of temporal sequence and one needs to deal with the out-of-sequence measurements (OOSMs) fusion problem. Additionally, origins of the received IR measurements are unknown, and the IR sensor can only measure the angle information from targets, which is highly nonlinear of the target kinematic state, both of these issues challenge the fusion system.

As shown in Figure 2, the proposed fusion mechanism includes four parts: angle only gating, pseudo measurement generation, nearest neighbor association as well as the IR information fusion, each part is described in detail in the rest of this subsection.

#### 2.3.1. Angle Only Gating

Since the IR sensor returns not only target measurements but also plenty of clutter measurements, in order to exclude clutter disturbance meanwhile reduce computation and storage burden, an elliptical angle only gating technique is implemented for each track to select feasible IR measurements for subsequent fusion.

(24) $${\left(z_{l,i}^{IR}-h^{IR}\left({\hat{x}}_{l|k},p_{l}^{IR}\right)\right)}^{T}{\left({\tilde{S}}_{l}\right)}^{-1}\left(z_{l,i}^{IR}-h^{IR}\left({\hat{x}}_{l|k},p_{l}^{IR}\right)\right)\leq \gamma ,$$

where γ is the gating probability that IR measurement falls inside the elliptical gate, the innovation covariance ${\tilde{S}}_{l}$ is obtained by

(25) $${\tilde{S}}_{l}={\tilde{H}}_{l}P_{l|k}{\tilde{H}}_{l}^{T}+R_{l}^{IR},$$

with ${\tilde{H}}_{l}$ denoting the IR measurement Jacobian matrix evaluated at ${\hat{x}}_{l|k}$, $p_{l}^{IR}$ is the IR sensor position at time $t_{l}$, $R_{l}^{IR}$ is the IR sensor measurements noise covariance. When $t_{l}<t_{k}$, the track kinematic state estimate ${\hat{x}}_{l|k}$ and its corresponding error covariance $P_{l|k}$ are obtained by

(26) $${\hat{x}}_{l|k}=F_{l,k}{\hat{x}}_{k|k},$$

(27) $$P_{l|k}=F_{l,k}P_{k|k}F_{l,k}^{T}+Q_{l,k},$$

where $F_{l,k}$ denotes the backward state transition matrix from time $t_{k}$ to $t_{l}$, $Q_{l,k}$ is the backward state process noise between time $t_{k}$ to $t_{l}$. When $t_{l}=t_{k}$, one has ${\hat{x}}_{l|k}={\hat{x}}_{k|k}$ and $P_{l|k}=P_{k|k}$. After gating, a subset of IR angle only measurements are selected and denoted as $z_{l}^{IR}$, with cardinality ${\tilde{m}}_{k}$.

#### 2.3.2. Pseudo Measurement Generation

As IR measurements are highly nonlinear and during conversion process multiple biases are added, therefore, pseudo measurements are generated based on IR angle only measurements and the track estimates in order to reduce any bias introduced. Here, pseudo measurements are defined in terms of the *i*th measurement of $z_{l}^{IR}$, that is, $z_{l,i}^{IR}={\left[\eta_{l,i}^{IR}\;\varepsilon_{l,i}^{IR}\right]}^{T}$, and the track kinematic state estimate ${\hat{x}}_{l|k}$ and its corresponding error covariance $P_{l|k}$. Let a = $\sin\eta_{l,i}^{IR}$, b = $\cos\eta_{l,i}^{IR}$, c = $\sin\varepsilon_{l,i}^{IR}$ and d = $\cos\varepsilon_{l,i}^{IR}$, then the pseudo measurement is defined as,

(28) $$y_{l,i}=H_{l,i}^{s}\left(\eta_{l,i}^{IR}\;\varepsilon_{l,i}^{IR}\right){\hat{x}}_{l|k}\overset{\Delta }{\;=}\left(\begin{array}{c} y_{s}1\\ y_{s}2 \end{array}\right),$$

where

(29) $$H_{l,i}^{s}=\left[\begin{array}{cccccc} a & -b & 0 & 0 & 0 & 0\\ cb & ca & -d & 0 & 0 & 0 \end{array}\right]$$

(30) $${\hat{x}}^{\tau }=y_{k|k}^{\tau }\left(1,1\right)\;;$$

(31) $${\hat{y}}^{\tau }=y_{k|k}^{\tau }\left(2,1\right)\;.$$

To form pseudo measurement ($y_{s}$), the approach used here takes actual measurements ($\eta_{IR},\varepsilon_{IR}$) and combines it with physical constraints (i.e., tan(η)=y/x). This pseudo measurement is the measurement input to tracking system that will lead to more accurate tracking. Thus, for scan *k*, using the measured angles [$\eta_{IR}\left(k\right),\varepsilon_{IR}\left(k\right)$] for that scan and the state predictions $\hat{x}\left(k|k-1\right)$, a measurement residual (${{\tilde{y}}_{s}}^{\tau }$) can be defined as,

(32) $${{\tilde{y}}_{s}}^{\tau }=0-{y_{s}}^{\tau }=-\left[\begin{array}{c} a{\hat{x}}^{\tau }-b{\hat{y}}^{\tau }\\ cb{\hat{x}}^{\tau }+ca{\hat{y}}^{\tau }-d{\hat{z}}^{\tau }. \end{array}\right]$$

The standard KF is used for state estimate given the measurement residual Equation (39). The measurement matrix (*H*) in standard KF is replaced by $H_{s}$. A pseudo measurement covariance matrix ($R_{s}$) is defined using the original angle measurement noise covariance matrix (*R*) and transformation matrix (*G*) and can be written as,

(33) $$R_{l,i}^{s}=GRG^{\prime },$$

where,

(34) $$G=\left[\begin{array}{cc} g_{11} & g_{12}\\ g_{21} & g_{22} \end{array}\right]$$

(35) $$g_{11}=\frac{\partial y_{s}^{\tau }1}{\partial \eta_{IR}^{\tau }}=b{\hat{x}}^{\tau }+a{\hat{y}}^{\tau }$$

(36) $$g_{12}=\frac{\partial y_{s}^{\tau }1}{\partial \varepsilon_{IR}^{\tau }}=0$$

(37) $$g_{21}=\frac{\partial y_{s}^{\tau }1}{\partial \eta_{IR}^{\tau }}=-ca{\hat{x}}^{\tau }+cb{\hat{y}}^{\tau }$$

(38) $$g_{22}=\frac{\partial y_{s}^{\tau }1}{\partial \eta_{IR}^{\tau }}=db{\hat{x}}^{\tau }+da{\hat{y}}^{\tau }+c{\hat{z}}^{\tau }$$

(39) $$R=\left[\begin{array}{cc} \sigma_{\eta_{{}_{IR}}}^{2} & 0\\ 0 & \sigma_{\varepsilon_{{}_{IR}}}^{2}. \end{array}\right]$$

$\sigma_{\eta_{{}_{IR}}}^{2},\sigma_{\varepsilon_{{}_{IR}}}^{2}$ are angle measurement error variances. Measurements are linearized by converting them to pseudo measurements and a measurement noise covariance matrix is introduced which depends on the state estimate and the measurements themselves. Measurement matrix ($H_{s}$) and measurement noise covariance matrix ($R_{s}$) are function of current measurements. By using the predicted measurements formed from the state predictions in $H_{s}$ and *G*, any coupling is eliminated. KF is applied for pseudo measurements to evaluate state estimate ($y_{{}_{2}k}$, $P_{{}_{2}k}$) and prediction ($y_{{}_{2}k|k-1}$, $P_{{}_{2}k|k-1}$). The resulting measurements are now expressed in 3-dimensional Cartesian coordinates and are fed to the NN algorithm for association with the proper track.

#### 2.3.3. Nearest Neighbor Association

The nearest neighbor [37] technique is most widely used for measurement association due to its low computational complexity and acceptable performance. Therefore, the nearest neighbor association is utilized here to obtain the best pseudo measurement for track fusion. The technique selects only one pseudo measurement which is nearest to the position estimate ${\hat{x}}_{l|k}^{p}$, denoted as $y_{l,i}$, along with its noise covariance $R_{l,i}^{s}$ claculated in the previous step. The gating and selection procedure can be expressed mathematically as,

(40) $$i^{*}=\arg\min_{1\leq i\leq {\tilde{m}}_{k}}{\left(y_{l,i}-{\hat{x}}_{l|k}^{p}\right)}^{T}{\left(S_{l,i}^{s}\right)}^{-1}{\left(y_{l,i}-{\hat{x}}_{l|k}^{p}\right)}^{T},$$

with the pseudo measurement innovation covariance equals to

(41) $$S_{l,i}^{s}=H_{l,i}^{s}P_{l|k}^{p}{\left(H_{l,i}^{s}\right)}^{T}+R_{l,i}^{s},$$

where, ${\hat{x}}_{l|k}^{p}$ and $P_{l|k}^{p}$ are the position component of the track estimate and its corresponding error covariance a time $t_{l}$, respectively.

#### 2.3.4. Ir Information Fusion

After nearest neighbor association, the associated IR pseudo measurement $\left(y_{l,i},R_{l,i}^{s}\right)$ is then utilized to fuse with the track estimate $\left\{\left({\hat{x}}_{k|k},P_{k|k}\right)\right\}$ at time $t_{k}$. Based on the time stamp of pseudo measurement $y_{l,i}$, there exist two possibilities about its identity, it could be ISM when $t_{l}=t_{k}$, and OOSM when $t_{l}<t_{k}$. Consequently, two different fusion strategies are carried out here. When $y_{l,i}$ is an ISM, the Kalman filter is implemented to update the track kinematic state estimate using $y_{l,i}$ by

(42) $$\left[{\hat{x}}_{k|k}\left(f\right),P_{k|k}\left(f\right)\right]=KF_{U}\left({\hat{x}}_{k|k},P_{k|k},y_{l,i},R_{l,i}^{s},H_{l,i}^{s}\right).$$

When $y_{l,i}$ is an OOSM, the Bl1 algorithm is deployed to to update the track kinematic state estimate using $y_{l,i}$. The Bl1 algorithm is a sub-optimal approach, it ignores the retrodicted process noise, by ignoring the process noise thus providing an approximate solution. However, multi-step lag problem is solved in single step using retrodiction based approach. The retrodicted state from current state at time $t_{k}$ to $t_{d}$ is represented as ${\hat{x}}_{l|k}^{B}={\hat{x}}_{l|k}$, with ${\hat{x}}_{l|k}$ calculated in Equation (26), associated covariances with retrodicted state are defined as,

(43) $$P_{vv}^{B}=Q_{l,k},$$

(44) $$P_{xv,i}^{\tau B}=Q_{k,d}-P_{{}_{2}k|k-l}^{\tau }{\left(S_{k}^{*}\right)}^{-1}Q_{k,d}.$$

The associated covariance with the retrodicted measurement will be defined as,

(45) $$S_{d}^{\tau B}=H_{d}^{ir}\left(Y_{{}_{2}k,i}^{\tau }\right)P_{d|k}^{\tau B}{\left(H_{d}^{ir}\left(Y_{{}_{2}k,i}^{\tau }\right)\right)}^{T}+R_{d}^{ir}.$$

At time *k* the covariance between current state and retrodicted measurement is expressed as,

(46) $$P_{xz}^{\tau B}=\left[P_{{}_{2}k,k}^{\tau }-P_{xv,i}^{\tau B}\right]F_{d,k}^{T}{\left(H_{d}^{ir}\left(Y_{{}_{2}k,i}^{\tau }\right)\right)}^{T}.$$

To update the state, gain is calculated by the equation,

(47) $$W_{d,k}^{\tau B}=P_{{}_{xz}}^{\tau B}{\left(S_{d}^{\tau B}\right)}^{-1}.$$

The OOSM $z_{d}$ is used to update the state estimate’s current state by mathematical equation as,

(48) $$Y_{k|d}^{\tau B}=Y_{{}_{2}k,i}^{\tau }+W_{d,k}^{B}\left[z_{d}-z_{d|k}^{B}\right].$$

The predicted OOSM $z_{d|k}^{B}$ is defined as,

(49) $$z_{d|k}^{B}=H_{d}^{ir}\left(Y_{{}_{2}k,i}^{\tau }\right)Y_{k|d}^{\tau B}.$$

With updated state, $Y_{k|d}^{\tau B}$, the associated covariance can be expressed as,

(50) $$P_{k|d}^{\tau B}=P_{k|k}^{\tau }-P_{zz}^{\tau B}{\left(S_{d}^{\tau B}\right)}^{-1}{\left(P_{zz}^{\tau B}\right)}^{T}.$$

The superscript “B” is used for Bl1 algorithm, the innovation covariance of OOSM is calculated by

(51) $$S^{\tau }{=H(P}_{k|k-1}^{\tau })H^{T}+R$$

with the covariance computed in the Bl1 algorithm.

(52) $$S_{d}^{\tau }=H_{d}^{ir}\left(Y_{{}_{2}k,i}^{\tau }\right)P_{d|k}^{\tau }{\left(H_{d}^{ir}\left(Y_{{}_{2}k,i}^{\tau }\right)\right)}^{T}+R_{d}^{ir}$$

and data association predicted measurement for IR,

(53) $$Y_{k|k-1}^{\tau }=F_{k}Y_{k-1|k-1}^{\tau }+v_{k}$$

is replaced by the predicted OOSM;

(54) $$z_{d|k}=H_{d}^{ir}\left(Y_{{}_{2}k,i}^{\tau }\right)Y_{d|k}^{\tau }.$$

### 2.4. Track Management and Output

After fusion with the IR sensor information, a set of improved track state estimates have been obtained as $\left\{P\left(\chi_{k}|Z^{k,r},Z^{l,IR}\right),\;\left({\hat{x}}_{k|k}\left(f\right),P_{k|k}\left(f\right)\right)\right\}$. In which the fused PTE $P(\chi_{k}|Z^{k,r},Z^{l,IR})$ is utilized as a track quality measure for track management. More specially, if the fused PTE exceeds a predefined confirmation threshold, this track is upgraded to a confirmed status which indicates it is following the target of interest and thus maintained to be confirmed. A confirmed track may become false track and is terminated if its PTE falls below a predefined termination threshold, this may happen if the confirmed track is misled to follow any clutter or targets of non-interest. Additionally, a tentative track may straightforwardly become false track in a few scans after initialization. Once a track is declaimed to be a false track, it is deleted from memory. As a result, the fusion system only outputs the kinematic states of the confirmed tracks at each time.

## 3. Simulation Study

In target tracking applications, data association is used to deal with clutter. In this research, the IPDA algorithm is modified to incorporate OOSMs using a sub-optimal approach. Results are computed for single sensor approach, fusion of sensors in cluttered environment (In sequence measurements) and fusion of sensors in cluttered environment with OOSMs. Results are compared for 250 Monte Carlo runs with each run having 58 scans. All the algorithms were implemented in MATLAB^®^ R2014a on system with Intel^®^ Core^TM^ i7-2600, 3.40 GHz processor, 8 GB memory and Windows^®^ 7 platform.

The performance comparison is done in terms of RMSE, PTE and FTD. These results are computed for a range of probabilities of detections that is, 0.6, 0.7, 0.8, and 0.9. The $P_{D}$ defines percentage of measurements availability for tracking process, if $P_{D}$ = 0.6 then only 60 percent of measurements are available for processing. RADAR and IR sensors are used for tracking, both sensors are located along z axis at [0, 0, 10 m] and [0, 0, 10.5 m],respectively. Initial position of target is [0, 30,000 m] with a uniform velocity of [0, −1000 m/s] along *x* and *y*-axis respectively . The uncertainty in the target measurements is expressed in terms of standard deviation, 5 m in each axis for the case of RADAR and 220 mRad in azimuth and elevation angles for the case of IR. Sampling time is 0.5 s and both sensors are assumed to be perfectly synchronized. Clutter measurement density is uniform and equals to $5\times 10^{-5}$/scan/m^2^.

Linear KF is used for estimation of state in the case of RADAR. RADAR measurements are first converted to Cartesian coordinate system, then KF is applied to update the state estimate. To update the IR information EKF is used, which uses estimate of KF as a posterior.

The euclidean distance is calculated between the actual track and the estimated track, if the calculated euclidean distance is less than a predefined maximum threshold then the track is considered a true track. For other track maintenance based statistics, different terms are used which are defined next. The nCases are defined as the total number of cases of a target being followed by a confirmed track at scan equal to 50 percent of the total scans. The nOK is defined as the total number of cases of a track still following the original target at scan equal to 80 percent of the total number of scans. The nResults is defined as total number of cases of a target being followed by a confirmed track at the last scan. Number of CFTs is also used as a performance metric of the proposed algorithm.

These stats and the number of CFTs show the overall performance of the proposed algorithm, which is compared for multiple probabilities of detection and for single sensor case as well.

Comparison of RADAR only and RADAR/IR system with 250 Monte Carlo runs is provided in terms of RMSE of position and velocity. Results are evaluated for the same CFTs, that is, CFT = 7 for $P_{D}$ = 0.9 for single sensor approach and multiple sensor (In sequence approach and OOSM) then the same thresholds are used in simulations of all $P_{D}$s.

The lower threshold (inital value of PTE) is kept the same for all three approaches, that is, 0.0005, while upper threshold (value of PTE for track confirmation) is set where 7 CFTs are observed in each algorithm. The values of upper thresholds are 0.8, 0.999, 0.997 for single sensor, multiple sensor (in sequence) and proposed algorithm, respectively.

**Figure 3:**
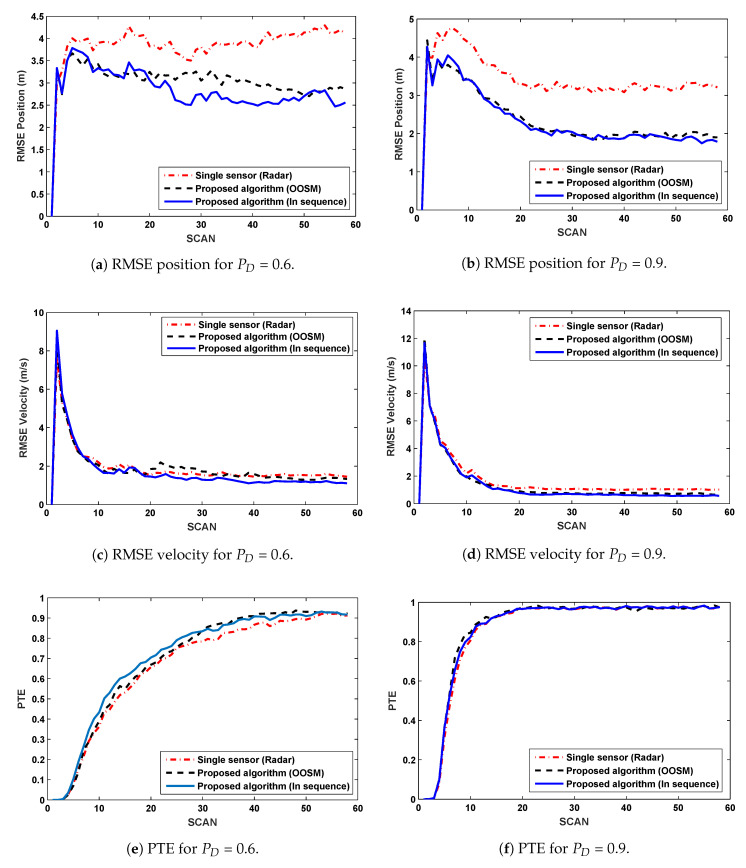
Comparison of RMSEs and Probability of Track Existence (PTE) for $P_{D}=0.6$ (**Left**) and 0.9 (**Right**).

The results for $P_{D}$ = 0.6 are presented in Figure 3a,c,e, it is evident from RMSE of position and velocity that minimum RMSE is for proposed algorithm with in sequence measurements, for OOSMs proposed algorithm’s RMSE is slightly high as compare to in sequence which is expected because sequential measurements are not available for estimation. RMSE of single sensor is worst as compared to multi-sensor approach. Algorithm used for single sensor approach is same as for the other two cases, the only difference is number of sensors. In figures it is observed that PTE is not much improved but it is a bit better in the case of multi-sensor approach although difference is not that considerable.

Figure 3b,d,f, show simulation results for $P_{D}$ = 0.9, almost same RMSE is observed for in sequence and OOSM approach. It can be seen that the RMSE performance for position and velocity increases with the proposed algorithm. The performance for in sequence as well as the OOSM case is better than the single sensor approach, whereas the PTE is slightly better than that of the single sensor approach.

**Figure 4:**
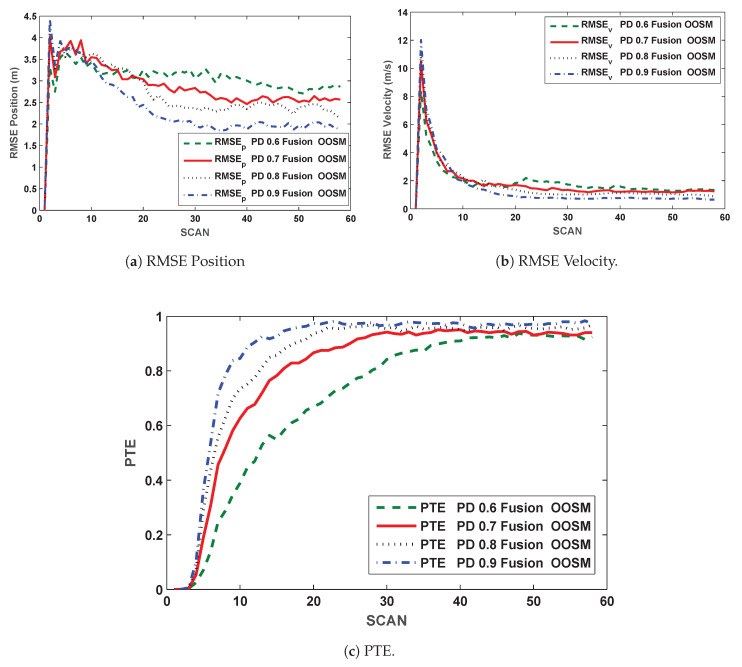
Comparison of RMSEs and PTE for multiple $P_{Ds}$ (Proposed Algorithm).

Comparison for multiple $P_{D}$s are presented for the proposed algorithm in terms of RMSE and PTE in Figure 4a–c. It is observed that performance improves as the $P_{D}$ improves that is, more estimation errors are observed for lower $P_{D}$s, which was the expected result.

Computational time for each run of the single sensor approach and the proposed algorithm is 178.9 and 169.6 ms, respectively. This was opposite to what was expected initially. Upon further investigation, it was found that the size of the validation gate reduces when multi-sensor fusion is performed due to the more accurate IR measurements. Since the number of measurements falling in each validation gate are reduced, therefore, the execution time is also reduced due to less number of operations in association as well as filtering. Equivalent measurements are estimated using Bl1 algorithm for 3 lag case, due to complexity of Bl1 algorithm increase in computational time was expected but redundancy provided by multiple sensor measurements and clutter rejection optimized it.

**Figure 5:**
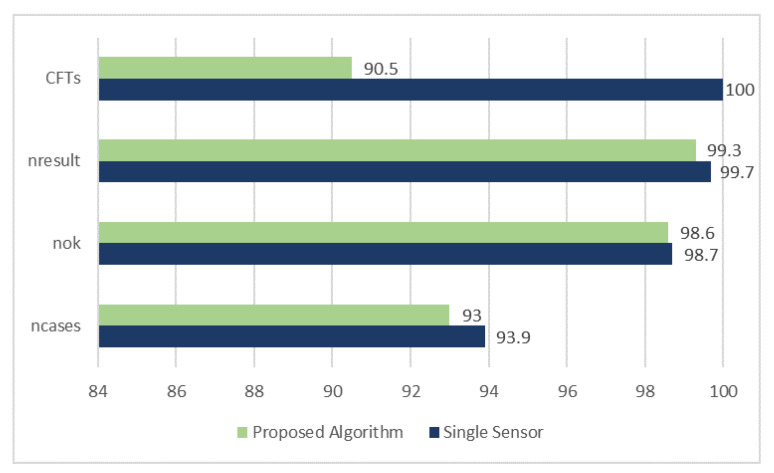
Comparison of accumulated CFTs and Statistics Percentage (for same CFTs at $P_{D}$ = 0.9).

Figure 5 shows comparison of normalized accumulated CFTs, CFT rate is high for single sensor approach as compared to the proposed algorithm.

Track retention Statistics are also presented in Figure 5, it shows approximately equivalent statistics for proposed algorithm as well as the single sensor approach. The single sensor approach is slightly better in terms of track retention as compared to the proposed algorithm at the cost of increased RMSE in the estimated target state dynamics and the number of confirmed false tracks.

## 4. Results and Discussions

Multiple sensor target tracking is implemented with few modifications in IPDA to improve the results in terms of RMSE of position and velocity. Results for single sensor and multiple sensor scenarios (both in sequence and out of sequence) are compared in Figure 3. It is evident from the results that multiple sensor approach improves the position and velocity estimate in comparison with the single sensor approach, to be exact, the position estimate is improved by 25% and 45% for $P_{D}=0.9$ and $P_{D}=0.6$, respectively. When the same approach is implemented for OOSM problem, the performance is slightly compromised in comparison with the in-sequence measurements but the performance is better than the single sensor approach. In case of OOSM, performance is compromised in terms of RMSE because equivalent state is estimated backward due to delayed measurements while in-sequence measurements are processed sequentially. The results of the proposed algorithm for OOSM are compared in Figure 4 for multiple $P_{D}$s. From these comparisons, it is evident that the performance of the algorithm improve as the $P_{D}$ improves. Normalized accumulated CFTs (for same CFTs at $P_{D}$ = 0.9) are compared for single sensor approach and the proposed algorithm in Figure 5, the proposed algorithm shows an improved CFT rate, where as single sensor approach shows better performance in terms of track retention.

The track retention statistics for the proposed algorithm are compared with the one proposed in Reference [3]. The algorithm was re-simulated in the current scenario with similar noise statistics as used in the simulation of proposed algorithm for a fair comparison. The results depicted in Table 1 clearly depict the superiority of the proposed algorithm in terms of track retention and FTD statistics at the cost of greater execution time, which, was obvious due to the choice of data association algorithm. The execution time will have implications for any real-time application of the algorithm, however, the results show that the proposed algorithm is suitable for such applications in the current form. Generally, when implemented on a hardware platform for real-time applications, algorithms are further optimized to use the available hardware resources more efficiently, this results in further reduction of the execution time.

While the algorithm is designed to perform efficiently for most of the scenarios, tracking performance will degrade in scenarios with higher clutter density at the IR sensor. This drawback in the algorithm is the target of a future research, in which we plan to implement a more advanced data association algorithm on the secondary tracker instead of the NN approach. Another important and challenging modification to this algorithm, which we plan to include in a future research, is to modify the existing algorithm for a multiple target tracking scenario.

## 5. Conclusions

This paper considers the fusion of RADAR and IR sensor data by incorporating OOSM in cluttered environment. A framework is developed for tracking the target accurately while providing redundancy to the system. A novel approach for incorporating OOSM is implemented in the framework of IPDA to handle the target tracking problem in cluttered environment. The proposed algorithm is evaluated for multiple sensor data fusion for both in-sequence and OOSM scenario. An improved estimation and tracking performance is observed in terms of RMSE of position/velocity and FTD when compared to other algorithms. The proposed algorithm for in sequence and OOSM case gives superior performance as compared to the single sensor approach in terms of RMSE by aprroximately 25%. In the case with less observable target that is, low $P_{D}$, FTD and track maintenance statistics are also better as compared to the single sensor approach where as the RMSE is superior by almost 45%. The proposed algorithm outperforms the technique used in Reference [3] in terms of track retention, the number of false tracks and the number of lost tracks by 11.3%, 10.5% and 11.3%, respectively.

## Figures and Tables

**Table 1:** Comparison of Track retention statistics for proposed algorithm and algorithm in Reference [3] for OOSM in cluttered environment.

Techniques	nResults %	nOk %	nCases %	CFT %	nLost %	Execution Time/Run (s)
**Algorithm in Reference [3]**	88	-	-	13.3	12	12.5 m
**Our Algorithm**	99.3	98.6	93	2.8	0.7	169.59 m
